# Validation of automated image co-registration integrated into in-house software for voxel-based internal dosimetry on single-photon emission computed tomography images

**DOI:** 10.1590/0100-3984.2022.0096

**Published:** 2023

**Authors:** André Luiz Alberti Leitão, Uysha de Souza Fonda, Carlos Alberto Buchpiguel, José Willegaignon, Marcelo Tatit Sapienza

**Affiliations:** 1 Centers for Radiology and Nuclear Medicine - Centro Médico, Brasília, DF, Brazil; 2 Hospital das Clínicas - Faculdade de Medicina da Universidade de São Paulo (HC-FMUSP), São Paulo, SP, Brazil; 3 Department of Radiology and Oncology - Faculdade de Medicina da Universidade de São Paulo (FMUSP), São Paulo, SP, Brazil; 4 Department of Nuclear Medicine - Instituto do Câncer do Estado de São Paulo (Icesp), São Paulo, SP, Brazil

**Keywords:** Dosimetry, Dose-response relationship, radiation, Tomography, emission-computed, single-photon, Image processing, computer-assisted, Dosimetria, Relação dose-resposta à, radiação, Tomografia computadorizada de emissão de fóton único, Processamento de imagem assistida por computador

## Abstract

**Objective:**

To develop an automated co-registration system and test its performance, with and without a fiducial marker, on single-photon emission computed tomography (SPECT) images.

**Materials and Methods:**

Three SPECT/CT scans were acquired for each rotation of a Jaszczak phantom (to 0°, 5°, and 10° in relation to the bed axis), with and without a fiducial marker. Two rigid co-registration software packages-SPM12 and NMDose-coreg-were employed, and the percent root mean square error (%RMSE) was calculated in order to assess the quality of the co-registrations. Uniformity, contrast, and resolution were measured before and after co-registration. The NMDose-coreg software was employed to calculate the renal doses in 12 patients treated with ^177^Lu-DOTATATE, and we compared those with the values obtained with the Organ Level INternal Dose Assessment for EXponential Modeling (OLINDA/EXM) software.

**Results:**

The use of a fiducial marker had no significant effect on the quality of co-registration on SPECT images, as measured by %RMSE (*p* = 0.40). After co-registration, uniformity, contrast, and resolution did not differ between the images acquired with fiducial markers and those acquired without. Preliminary clinical application showed mean total processing times of 9 ± 3 min/patient for NMDose-coreg and 64 ± 10 min/patient for OLINDA/EXM, with a strong correlation between the two, despite the lower renal doses obtained with NMDose-coreg.

**Conclusion:**

The use of NMDose-coreg allows fast co-registration of SPECT images, with no loss of uniformity, contrast, or resolution. The use of a fiducial marker does not appear to increase the accuracy of co-registration on phantoms.

## Abstract (pt)

**Objetivo:**

Desenvolver corregistro automático e testar seu desempenho com ou sem marcador fiducial em imagens de tomografia computadorizada de emissão de fóton único (SPECT).

**Materiais e Métodos:**

Três SPECT/CTs foram adquiridas para cada rotação de um simulador de Jaszczak em relação ao eixo da maca (0°, 5° e 10°), com e sem fiducial. Dois métodos de corregistro inelástico foram aplicados - SPM12 e NMDose-coreg -, e a porcentagem do erro quadrático médio (%RMSE) foi usada para analisar a qualidade do corregistro. Uniformidade, contraste e resolução foram medidos antes e após o corregistro. NMDose com corregistro automático foi usado para calcular a dose renal de 12 pacientes tratados com ^177^Lu-DOTATATE e comparado com OLINDA/EXM.

**Resultados:**

A marcação fiducial não modificou a qualidade do corregistro das imagens SPECT, medida pela %RMSE (*p* = 0,40). Não houve impacto na uniformidade, contraste e resolução após o corregistro de imagens adquiridas com ou sem fiduciais. Aplicação clínica preliminar mostrou tempo total de processamento de 9 ± 3 min/paciente para NMDose e 64 ± 10 min/paciente para OLINDA/EXM, com alta correlação entre ambos, apesar de menor dose renal em NMDose.

**Conclusão:**

NMDose-coreg permite o corregistro rápido de imagens SPECT, sem perda de uniformidade, contraste ou resolução. O uso da marcação fiducial não aumentou a precisão do corregistro em fantomas.

## INTRODUCTION

Internal dosimetry can help personalize the radionuclide administration protocols for the treatment of various tumors, by estimating the radiation dose delivered to the tumor and critical organs^(1)^. However, there are still limitations to the implementation of dosimetry in the clinical routine of therapeutic planning^(2,3)^. The absorbed fraction method proposed by the Medical Internal Radiation Dose (MIRD) Committee of the Society of Nuclear Medicine has gained wide acceptance as the standard method for performing internal dosimetry calculations^(4)^. Extension of the MIRD schema to the voxel level, based on voxel S value calculations, is described in MIRD pamphlet no. 23^(5)^. In recent years, there have been various studies and the development of commercial voxel-based internal dosimetry software, such as VRAK^(6)^, RAYDOSE^(7)^, VIDA^(8)^, VoxelMed^(9)^, and BIGDOSE^(10)^.

Voxel-based dosimetry is based on the integration of the activity over time in each voxel, rather than in source and target organs. A fundamental task in voxel-based dosimetry is the correct registration of images acquired at different intervals, so that each voxel corresponds to the same patient spatial coordinates at all time points^(11-13)^. Most software provides manual or rigid image registration, based on computed tomography (CT) or single-photon emission computed tomography (SPECT). Mismatches between SPECT and CT can affect CT-based registration and the quantitative estimation of activity for internal dosimetry^(14)^, a situation that can be avoided if registration is performed on SPECT images alone.

The use of radioactive fiducial markers, as previously proposed for registration of nuclear medicine images in scintigraphy^(15)^ and radiation therapy planning^(16)^, might improve the co-registration of SPECT images. Fiducial markers are also used in order to merge images acquired by different modalities.

The aim of this study was to develop an automated co-registration method and test its performance, with and without a fiducial marker, on SPECT images of a phantom. The registration method was integrated into our in-house software (NMDose-coreg) and applied for retrospective dosimetry in patients treated with ^177^Lu-DOTATATE.

## MATERIALS AND METHODS

The project was approved by the local research ethics committee (Reference no. 38519014.8.0000.0065; Record no. 882.641).

**Figure 1:**
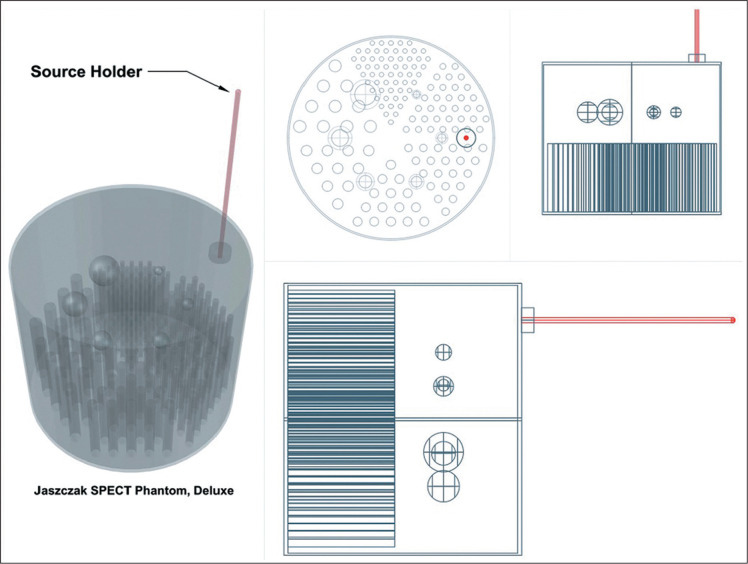
Phantom schematic and fiducial source holder.

To measure co-registration consistency with and without fiducial markers, SPECT/CT scans of a phantom Jaszczak DLX (Data Spectrum Corporation, Durham, NC, USA) (Figure 1) were acquired in a four-slice scanner (Infinia Hawkeye 4; GE Healthcare, Buckinghamshire, UK). The standard SPECT acquisition involved 120 angular projections over 360°, 500,000 counts per view, a low-energy high-resolution collimator, a zoom factor of 1.33, and a 128 × 128 matrix. The CT was performed with a tube voltage of 120 kVp, a tube current of 1.0 mAs, a pitch of 1.6, and a 512 × 512 matrix. The images were reconstructed by using the ordered subset expectation maximization iterative technique, together with a Hann Pre-Filter at a critical frequency of 1.56 cycles/cm, with attenuation correction measured by CT.

A solution of 740.0 ± 18.5 MBq of technetium-99m pertechnetate diluted in 6.1 L of water was used in order to fill the phantom and the 3.0-mL fiducial marker. Three SPECT/CT images were acquired for each clockwise rotation of the phantom in relation to the bed axis (0°, 5°, and 10°), with and without a fiducial marker; therefore, a total of 18 studies were performed.

### Image co-registration

Two co-registration software packages were employed: SPM12 and NMDose-coreg. The SPM12 program is an add-on for Matlab (MathWorks, Natick, MA, USA), developed in the Functional Imaging Laboratory of the Wellcome Centre for Human Neuroimaging at University College London^(17)^. The co-registration in SPM12 is based on the work of Collignon et al.^(18)^. When working with intramodality registration, as was done in the present study, iterative convergence of the image volumetric matrix is based on the entropy correlation coefficient or normalized cross-correlation. Before processing, the SPECT images had to be converted from Digital Imaging and Communications in Medicine (DICOM) format to Neuroimaging Informatics Technology Initiative (NIfTI) format. In the fiducial marker group, the image origin was manually set on the marker by using the SPM12 triangulation for automated registration. In the non-fiducial marker group, the origin was set in the middle of the phantom. The normalized cross-correlation method was applied, with an average distance of 4.0 mm between the sample points, smoothed with a Gaussian function with a full width at half maximum (FWHM) of 7 mm. All target SPECT scans (images rotated 5° and 10°) were corrected and resliced to the reference (0°) SPECT scan.

The NMDose-coreg program was developed in-house and applies minimization of the mean squared error after the translation and rotation of the images. The least-squares function approximates the intensity histogram distributed in the volume of the target image, in comparison with that of the reference image, with a gradient tolerance of 1.0 × 10^-4^ and a convergence tolerance of 1.0 × 10^-5^, in a maximum of 100 iterations.

### Co-registration quality

To assess the quality of the registration quality, we calculated the percent root mean square error (%RMSE) of the phantom, comparing the reference image r(x,y) with the registered image g(x,y)^(19)^. The RMSE measures the difference between the counts in each voxel before and after co-registration, and its relationship to the total SPECT counts gives the %RMSE:


$$RMSE=\sqrt{\frac{1}{n_{x}n_{y}}\sum_{0}^{n_{x}-1}\sum_{0}^{n_{y}-1}[r(x,y)-g(x,y){]}^{2}}$$

where *n_x_* and *n_y_* represent the position in the matrix in the image slice, *r*(*x,y*) is the image reference function and *g*(*x,y*) is the co-registered image function (*x* and *y* are the voxel coordinates in a given image slice).

The impact of image manipulation on uniformity and resolution was determined by following the International Atomic Energy Agency manuals, with the aid of the International Atomic Energy Agency-Nuclear Medicine Quality Control Toolkit plugin^(20)^, before and after co-registration with SPM12 and NMDose-coreg. Contrast was calculated in the slice containing the cold spheres, with automated detection of the minimum and mean activity in each of the spheres, compared with the activity in a user-defined area with uniform activity. The resolution was also estimated by fitting the count profile of the Jaszczak rods with multiple Gaussian functions and calculating the FWHM^(21)^.

Descriptive statistics were used in order to analyze %RMSE, uniformity, and resolution. One-way analysis of variance was used for comparisons among three or more groups, and unpaired Student’s t-test was used for comparisons between groups. The level of significance was set at 5%.

### Clinical testing (preliminary)

The NMDose-coreg program was integrated into the NMDose software package and used in order to calculate the renal doses in 12 patients treated with ^177^Lu-DOTATATE for neuroendocrine tumors. As previously described^(22)^, NMDose is an in-house software package. Its flow chart incorporates co-registration; activity integration; automated segmentation for bone and soft tissues; and absorbed dose calculation using dose-point kernel convolution for iodine-131, lutetium-177, or yttrium-90, resulting in a three-dimensional dose map recorded in DICOM format. The time-integrated activity per voxel is quantified by using the trapezoidal rule for the uptake period and a double-exponential fit for decay kinetics. The convolution utilizes a table of the values of the absorbed dose rate per unit of activity (S values) generated by DOSXYZnrc^(23)^. To differentiate S values between bone and soft tissue voxels, we used automated bone segmentation with a cutoff of 300 Hounsfield Units.

The SPECT/CT images were co-registered at four post-injection time-points: 1-2 h, 4-6 h, 24 h, and 240 h. The renal dose was calculated assuming the mean dose distribution in a volume of interest defined on the parametric dose map image, with a cutoff of 40% of the maximal kidney dose. All patients were part of a research project approved by the local research ethics committee, and the dosimetry procedure did not modify the treatment.

Time efficiency was determined by measuring the time elapsed between the co-registration step and the NMDose dose map assessment, which was then compared with that required for the handwork employed to calculate the dose using the Organ Level INternal Dose Assessment for EXponential Modeling (OLINDA/EXM) software on the same computer. We employed a desktop computer with the following configuration: Intel core i7-6700HQ CPU at 2.60 GHz; RAM of 32.0 GB (usable: 31.9 GB); 64-bit operating system, x64-based processor; GPU: NVIDIA GeForce GTX 980 M. The OLINDA/EXM software has been approved by the US Food and Drug Administration; it performs dose calculations and kinetic modeling for radiopharmaceuticals based on the user-provided biokinetics of the radiotracer in source and target organs, according to the MIRD formalism^(24)^. For the calculation using OLINDA/EXM, we manually measured the whole-body, kidney, liver, spleen, and bladder uptakes, adjusting the dimensions of critical organs by using a reference CT image. Pearson’s correlation coefficient and Bland-Altman plots were used in order to compare the renal doses estimated by NMDose-coreg with those estimated by OLINDA/EXM.

## RESULTS

### Image co-registration

**Figure 2:**
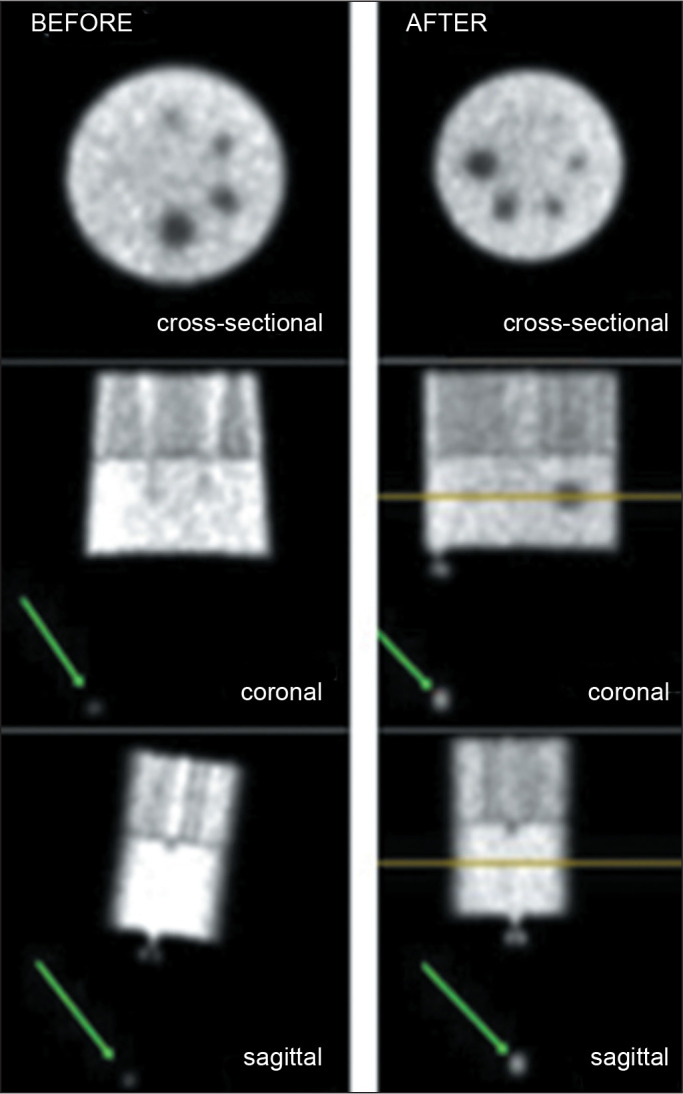
SPECT scans of the phantom with 10° rotation (target images) before and after co-registration with the unrotated SPECT scans (reference images) by NMDose-coreg. The arrows indicate the fiducial marker.

We acquired SPECT/CT scans of the phantom with and without a fiducial marker, with three acquisitions for each position of the phantom (0°, 5°, and 10°); therefore, a total of 18 studies were performed. Rotated (target) images were co-registered to the unrotated SPECT (reference) image with NMDose-coreg and SPM12, and visual analysis revealed good spatial registration, as shown in Figure 2.

### Co-registration quality

**Figure 3:**
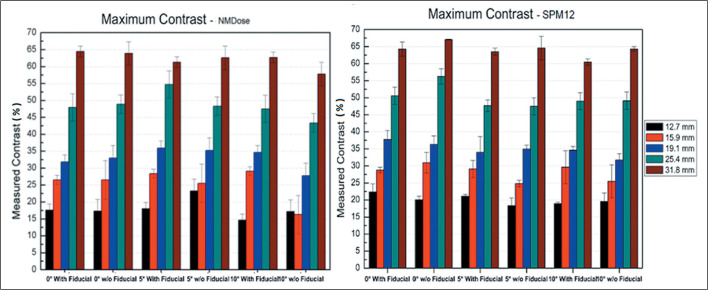
Maximum contrast variation with respect to angle rotation for co-registration by NMDose and SPM12.

Table 1 shows the %RMSE, uniformity, and resolution for both co-registration methods. Figure 3 shows the maximum contrast measurements. There was no statistically significant difference in %RSME between images acquired with and without a fiducial marker, whether processed by SPM12 (*p* = 0.48) or by NMDose-coreg (*p* = 0.40). There was also no significant difference between the two groups in terms of uniformity (*p* = 0.54) or resolution (*p* = 0.44).

**Table 1:** %RMSE, uniformity, and resolution for all images co-registered by SPM12 and NMDose-coreg, by group (based on the degree of rotation of the phantom and the presence or absence of a fiducial marker).

Image group	%RMSE		Uniformity		Resolution	
	SPM12	NMDose-coreg	SPM12	NMDose-coreg	SPM12	NMDose-coreg
0° with fiducial marker	-	-	4.9 ± 0.6	5.2 ± 0.8	12.6 ± 2.9	13.4 ± 3.0
0° without fiducial marker	-	-	6.7 ± 1.0	6.5 ± 0.5	11.7 ± 2.9	11.2 ± 0.4
5° with fiducial marker	6.46E-04	4.21E-04	5.3 ± 1.4	5.6 ± 0.6	11.5 ± 2.2	11.8 ± 0.7
5° without fiducial marker	6.85E-04	1.18E-03	5.5 ± 0.2	5.5 ± 0.6	12.5 ± 3.0	13.3 ± 1.8
10° with fiducial marker	6.60E-04	4.20E-04	5.8 ± 1.6	5.7 ± 1.7	13.3 ± 3.5	13.3 ± 1.5
10° without fiducial marker	4.21E-04	7.38E-04	5.0 ± 0.4	5.1 ± 0.6	13.8 ± 2.9	11.4 ± 1.3

### Clinical testing (preliminary results)

**Figure 4:**
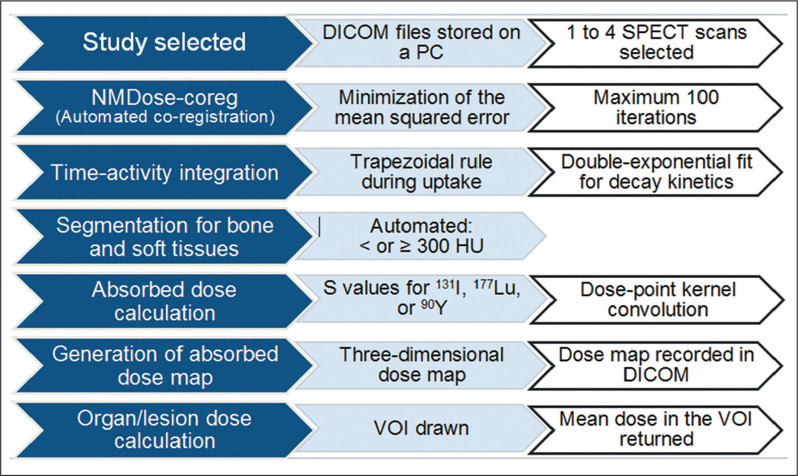
NMDose flow chart. ^131^I, iodine-131; ^177^Lu, lutetium-177; ^90^Y, yttrium-90; VOI, voxel of interest.

**Figure 5:**
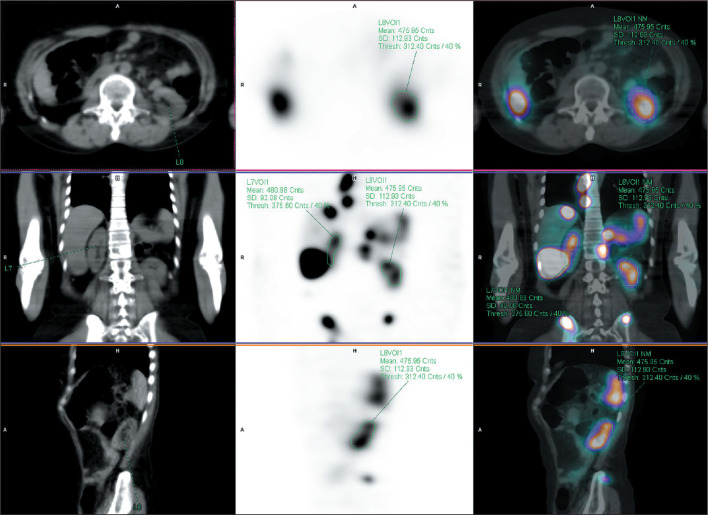
Parametric dose map obtained with NMDose-coreg: cross-sectional and coronal images fused with the CT image. A volume of interest was drawn by applying a cutoff of 40% of the maximum dose in the kidney.

**Figure 6:**
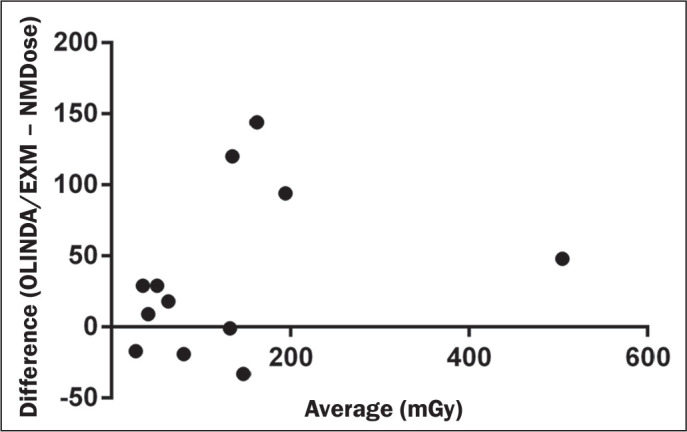
Bland-Altman plot of OLINDA/EXM versus NMDose-coreg in terms of the absorbed doses to the kidneys.

The NMDose-coreg was integrated into the NMDose flow chart (Figure 4), and the renal dose was calculated for each of the 12 patients (Figure 5). The mean ± standard deviation flow chart execution time was approximately 9 ± 3 min for NMDose and 64 ± 10 min for OLINDA/EXM. The mean renal dose calculated by NMDose-coreg was 113 ± 125 mGy, compared with 148 ± 141 mGy for OLINDA/EXM. The correlation coefficient for dose distribution was 0.92, with a significant difference between the two methods (*p* = 0.00003). The Bland-Altman plot comparing doses calculated by OLINDA/EXM and NMDose is shown in Figure 6.

## DISCUSSION

Image registration, matching organs or lesions with the same voxel coordinates across all images, is essential for voxel-based dosimetry. The use of fiducial markers to improve co-registration is not unanimously accepted, even in radiotherapy. Fiducial markers can be justified for prostate cancer radiotherapy planning, because the proximity of organs with physiological movement, such as the bladder and intestine, can result in displacement of the target^(25)^. For other targets in radiotherapy, the sub-millimeter resolution of CT provides the precision required for co-registration based only on the anatomical landmarks.

Images acquired by SPECT have much lower resolution than do those acquired by CT, the former having a resolution of approximately 10 mm, and fiducial markers might improve registration when SPECT/CT is not available or when a SPECT/CT mismatch is suspected. In the present study, we observed a high level of agreement between NMDose-coreg and SPM12, regardless of the use of fiducial markers. A critical note when using SPM12 is that, in our experience, the required process of transforming the image from DICOM format to NIfTI format can affect the signal amplitude and increase the count by up to 30 times over that obtained from an untransformed image. The difference originates from the misinterpretation of scale factors in converting the DICOM header to a NIfTI header, which is not critical for visual interpretation or even for relative measurements of the images but makes quantification unfeasible.

Among the studies performed without a fiducial marker, the average %RMSE was higher for those that were processed with NMDose-coreg (using the least-squares approach). However, there was no statistically significant difference in image fit between SPM12 and NMDose-coreg. The uniformity, contrast, and resolution of registered images remained similar to those of the reference images. A limitation of the co-registration technique implemented in this study is that it is rigid, with no changes in the intensity profile of each image during the translation and rotation in the three axes. The physical interpretation would be to consider the image as a non-deformable solid^(12)^. Whether performed manually or by computer, this approach disregards the movements of internal organs. Therefore, the exact spatial location of a mobile lesion (i.e., a tumor in the gut) can be difficult to register, resulting in underestimation of the calculated dose. However, it is an elegant solution for patient positioning and movement errors, automating a significant step that is quite time-consuming when performed manually.

Under the conditions studied, the automated object repositioning error was small, regardless of whether or not a fiducial marker was used. Decreasing the phantom signal strength information and maintaining or even increasing the activity of the fiducial marker could lead to different results, and the same could occur in clinical conditions with different distributions and count rates. Hence, we tested the method in a real clinical situation: NMDose-coreg implemented in NMDose and used in patients submitted to radionuclide therapy with ^177^Lu-DOTATATE. It should be noted that the phantom measurements were performed with technetium-99m pertechnetate, due to the greater availability of that radionuclide, whereas the patients were injected with lutetium-177. Although physical differences can theoretically affect the co-registration, visual analysis of the four sequential SPECT images acquired before and after co-registration confirmed that NMDose-coreg performed well.

Time efficiency was substantially better when NMDose was used than when OLINDA/EXM (the manual process) was used, the difference being approximately 711%. Although the doses calculated by the NMDose and OLINDA/EXM algorithms correlated well, there was a significant difference between the results, the doses estimated by NMDose being lower. In addition, the Bland-Altman analysis showed differences in estimated individual doses of up to 144%, well above the expected intra-method uncertainty of 10-20%^(26,27)^. However, other studies comparing dosimetry methods have also found large differences, in particular when voxel-based and organ-based dosimetry are compared. In a study involving six healthy volunteers, the mean ratio between of in the renal dose calculated with a voxel-based Monte Carlo (GATE) method and that calculated with the OLINDA/EXM algorithm was 1.48 ± 0.61, lower than the 2.08 ± 0.97 obtained when GATE was compared with the commercial voxel-based software STRATOS^(28)^. In addition, a direct comparison of five different commercial voxel-based dosimetry software platforms in two patients treated with ^177^Lu-DOTATATE showed a mean difference of 82% between the minimum and maximum estimated renal dose, the individual difference being 175% in the most discrepant case^(29)^. That difference could be attributed, in part, to inaccurate generalization of the patient geometry and actual target organ mass in OLINDA/EXM, as well as to the assumption that the activity is uniform in each manually segmented organ.

Given that OLINDA/EXM, despite the abovementioned criticisms, was employed as the standard for internal dose calculation in the present study, further evaluations are needed in order to identify the reasons for discrepancies before the software can be made available for research or clinical use. The programming code still lacks the implementation of contrast recovery and produces errors associated with the calibration factors. In addition to error propagation analysis, further program development and validation would require comparison with other voxel-based software in larger patient samples.

## CONCLUSION

The proposed method, based on minimization of the mean squared error function, allows fast, automated co-registration of SPECT images, without losses of uniformity, contrast, or resolution. Under the conditions studied, the use of a fiducial marker does not appear to increase the accuracy of co-registration on phantoms.

The integration of NMDose-coreg into NMDose makes it possible to estimate the renal radiation dose in patients undergoing therapy with ^177^Lu-DOTATATE, showing a high correlation with the standard method (OLINDA/EXM), despite the dose estimated by NMDose-coreg being lower than that estimated by OLINDA/EXM. For the NMDose-coreg software to be applicable in therapeutic planning, greater understanding of the causes of discrepancy in the dose calculation and further development of the program are necessary.

## Data Availability

NMDose is licensed under a Creative Commons Attribution Share-Alike (cc-by-sa) License. Codes are available at https://mednuclear.wixsite.com/dosimetria/projects.
